# Knowledge and practice of Essential Newborn Care among postnatal mothers in Mekelle City, North Ethiopia: A population-based survey

**DOI:** 10.1371/journal.pone.0202542

**Published:** 2018-08-22

**Authors:** Tirhas Asmelash Berhea, Abate Bekele Belachew, Girmatsion Fisseha Abreha

**Affiliations:** 1 Maternal and Child Health Department, Tigray Regional Health Bureau, Mekelle, Ethiopia; 2 Department of Biostatistics, School of Public Health, College of Health Sciences, Mekelle University, Mekelle, Ethiopia; 3 Department of Reproductive Health, School of Public Health, College of Health Sciences, Mekelle University, Mekelle, Ethiopia; Paediatric Centre of Excellence, ZAMBIA

## Abstract

**Background:**

In Ethiopia, neonatal mortality remains high and accounts for about half of the under-five mortality. However, there is limited data on the knowledge and practice of mothers about newborn care at the community level, particularly in urban settings. Therefore, this study aimed to assess knowledge and practice of mothers on Essential Newborn Care in urban communities.

**Methods:**

A population-based cross-sectional study was conducted in December 2016 in Mekelle City, Northern Ethiopia. A total of 456(weighted) postpartum mothers were included in this study. A three-stage cluster sampling was used to select the study subjects in which districts, Kebeles and respondents formed the first, second and third stage, respectively. Postnatal mothers were recruited from each cluster/Kebele until the required sample size was achieved. They were interviewed using a structured questionnaire. Mothers who responded correctly to at least 75% of the knowledge and practice questions were considered to have good knowledge and practice, respectively. Multivariable logistic regression was used to identify factors associated with the knowledge and practice of Essential Newborn Care.

**Results:**

In this study, 36.1% of mothers had good knowledge and 81.1% had a good practice on Essential Newborn Care. Newborn care practice was positively associated with those mothers who were educated [Adjusted Odds Ratio (AOR) = 1.94; 95% CI (1.07, 3.50)], counseled during delivery and postpartum [AOR = 4.97; 95% CI (1.93, 12.76)], who had good knowledge of newborn care [AOR = 2.32; 95%CI (1.18, 4.55)] and who had good knowledge of newborn danger signs [AOR = 2.43; 95%CI (1.21, 4.87)].

**Conclusions:**

A substantial number of postpartum mothers had poor knowledge and practice on Essential Newborn Care in Mekelle City. Therefore, improving quality and access to maternal health services and home visit using the urban health extension workers at the community level should be encouraged.

## Background

Despite the progress towards reduction of under-five mortality globally, newborn deaths have reduced at a slower rate [1]. About 6 million children die before their fifth birthday each year. About 5 million of these deaths occur in the first year of life and nearly 3 million die within the first 28 days of birth. This indicates that about 45% of under-five deaths and 60% of infant deaths are accounted for the neonatal mortality. Almost all (99%) of these neonatal deaths occur in low income and middle-income countries, with the highest rates occurring in Sub-Saharan Africa (29 deaths per 1000 live births in 2015). Majority of these deaths however are due to preventable causes [2–4].

In Ethiopia, among the 184,000 children under five who die, 87,000 are babies within the first four weeks of life [1]. Neonatal death accounts for 47% of all deaths in under-five children younger than five years of age. In 2015, 28 per 1000 live births died mainly due to bacterial sepsis of the newborn, birth asphyxia, perinatal respiratory disorder and respiratory distress related to prematurity [1, 5]. The risk of death is highest in the first 24 hours of life when more than half of deaths occur and about three–quarters of all neonatal deaths occur within the first week of life [6, 7]. It is estimated that about 75% of neonatal deaths could be avoided with simple and low-cost interventions [2]. The neonates are at risk for various health problems even though they are born with an average birth weight. Most of the health problems are life-threatening to the neonates. Therefore, they need optimal care for their survival. The WHO Essential Newborn Care (ENBC) recommendations are important to reduce the neonatal illness and deaths.

Ethiopia has made tremendous efforts in improving the quality and access to health service by implementing a health extension program [8]. However, attendance of skilled workers during delivery (16.4%) and postnatal care (13%) is still very low. Majority of mothers deliver at home in the presence of traditional birth attendants, which has resulted in many harmful traditional practices applied to the newborn baby by mothers and grandmothers [9, 10]. Babies receive little attention until the placenta is expelled. They may or may not be covered in cloth or dried [10]. No skin to skin contact is reported and newborns have often been placed slightly away from the mother’s side. Attendants are largely focused on the delivery of the placenta and the related well-being of the mother [10]. Insufficient knowledge of parents during this period could lead to parents’ confusion and decreased quality of care [11, 12] that in turn threaten the neonatal health and could even leads to neonatal mortality [13]. Therefore, assessing maternal knowledge and practice towards ENBC has valuable importance in the healthy development of the newborn. Moreover, there is limited information about postnatal mothers’ knowledge and practices on ENBC in Ethiopia, particularly in urban settings. Studies that assessed knowledge and practice of postnatal mothers towards newborn care in the country are mainly confined to rural settings that have a significantly different socio-demographic and economic status and access to health facilities. Moreover, despite the implementation of the Urban Health Extension program, a significant proportion (41.5%) of urban women still had a home birth [9], and neonatal deaths are even higher than the rate reported for rural settings [6]. Thus, the level of knowledge and practice and the associated factors could vary significantly across settings. Therefore, this study aims to determine the success of the urban health extension program. In addition, some key indicators of ENBC are not currently measured by routine surveys like the Demographic and Health Survey. Therefore, this study is aimed to investigate the mothers’ knowledge and practice towards ENBC practice in Ethiopia.

## Methods

### Study setting and design

This study was conducted in Mekelle City, the capital city of the Tigray regional state, Northern Ethiopia. The city is located 783 kilometers away from Addis Ababa, the capital of Ethiopia. It is comprised of seven districts (sub-cities). Each district (sub-city) is also comprised of five Kebeles (lower administrative units) and has an estimated population of 5000 in each Kebele.

A population-based cross-sectional study was conducted among permanent resident mothers in Mekelle city from December 1 to 30, 2016. Postnatal mothers within 42 days after their live birth were the actual participants of this study. A sample size was estimated using Epi-info version 7 software, using the prevalence of good knowledge of neonatal danger signs [14] (18.2%), 95% of confidence, 5% margin of error. By considering 10% non-response and a design effect of 1.5, the final sample size of 378 was arrived at. Mothers were selected using multi-stage cluster sampling technique. First, four districts of the total of seven were selected by lottery method. Then proportional allocation was used to determine the sample size per district utilizing a one month report of postnatal mothers. Accordingly, a total of 99, 108, 111 and 60 samples were allocated to Ayder, Hadnet, Hawelty and Semen sub-cities, respectively. Then, each district was clustered by Kebele. Three Kebeles (clusters) were selected from each district randomly, therefore a total of 12 Kebeles were included in this study. The allocated sample for each district was divided equally for the selected three Kebeles. Then, eligible postnatal mothers were interviewed until the required sample size was attained.

### Study variables and measurements’

Data was collected using a structured questionnaire, which was adapted from various literatures [15–19]. The questionnaire was designed to gather information regarding the mothers’ socio-demographic characteristics, reproductive and obstetric history and knowledge and practice of the WHO Essential Newborn Care guidelines. To ensure data quality, the questionnaire written in English was translated into the local language (Tigrigna) and back to English and pretested on postnatal mothers selected from similar setting but out of the study area. Face to face interview was conducted by five trained data collectors who are female diploma nurses with two supervisors. Day to day on-site supervision during the whole period of data collection was done.

Knowledge and practice of postnatal mothers towards ENBC were the two main outcome variables in this study. Knowledge was assessed using a total of 18 “Yes/No” questions on various aspects of newborn care (e.g. Knowledge regarding immunization, thermoregulation, breastfeeding, cleanliness and umbilical cord care, and eye care were assessed). The values were coded as “1 = Correct response (consistent with WHO ENBC guidelines) and 0 = Incorrect response (inconsistent with WHO ENBC guidelines)”. Finally, a composite variable from these questions was generated to categorize mothers as having “Good/poor knowledge”. Mothers who responded correctly to at least 75% of questions were categorized as having good knowledge.

The newborn danger signs were assessed using a series of nine close ended questions and a composite variable was generated from these questions to categorize mothers as having “Good/poor” knowledge. Accordingly, mothers who correctly responded to at least 3 questions were categorized as having a good knowledge of newborn danger signs [14].

The practice was assessed using a series of 8 close ended questions (e.g. Practice on thermoregulation, breastfeeding, cleanliness and umbilical cord care, and eye care). Then, a composite variable from these questions was generated to categorize mothers as having “Good/poor practice”. Accordingly, mothers who responded correctly to at least 75% of questions were categorized as having a good practice.

### Statistical analysis

Data obtained using the questionnaire were entered into Epi-data version 3.1, and transported to SPSS software version 20 for cleaning and statistical analysis. Frequency and percentages were used to summarize the data. A multivariable logistic regression model was fitted to identify the socio-demographic, reproductive and obstetric history of the mothers and exposure to information or education about ENBC related factors associated with knowledge and practice of mothers towards ENBC. The degree of association between independent and dependent variables was assessed by using odds ratio at 95% level of confidence. The clustering nature of the survey might lead to small standard errors that may result in false positives. Therefore, the robust standard errors estimation was used to account for cluster design so as to see the valid relationship between the outcomes and exposure variables. To adjust for unequal probability of selection of samples from the districts, sampling weights were used to compute descriptive statistics. Therefore, the estimates are reliable and can be inferred to the mothers of the study area.

### Ethical approval

The Ethical approval was granted by the Institutional Review Board of the College of Health Sciences, Mekelle University [protocol approval-EBC-06130/2016]. Before collecting the data, informed verbal consent was obtained from each study subject. The data collectors explained an information sheet prepared in the participant’s local language [Tigrigna] and asked for the consent of the participant. The participation in the study was fully voluntary. Provided that the data collection process did not involve more than minimal risk to the participants (as there was no specimen/blood sample collection) and given that the majority of the study participants could not read and write, a written consent was waived by the Institutional Review Board. All information gathered was anonymous to protect confidentiality. To ensure the privacy of study participants, all interviews were conducted at their home in the private area.

## Results

### Socio-demographic characteristics of study participants

In this study, 373 (n = 456 –weighted) eligible mothers were participated. The mean and standard deviation of the age of mothers were 28 and 5.5 years, respectively. The majority of the study participants were Orthodox Christian, 375(82.2%). Employed women accounted for 78 (17.2%). One hundred twenty-seven (34%) husbands were educated diploma and above. One hundred forty six (32.1%) earned ≤ 1500 Ethiopia Birr per month and 47 (10.7%) of the participants residence was located more than 30 minutes away from a health facility (Table 1).

**Table 1:** Socio-demographic characteristic of the respondents in Mekelle City, Tigray, Ethiopia, 2016 (n = 456).

Socio-demographic variables	Frequency	Percentage
**Religion**		
Orthodox	375	82.2
Muslim	67	14.6
Catholic and Protestant	14	3.2
**Marital status**		
Married	414	90.9
Single	26	5.6
Divorced/Widowed	16	3.5
**Mothers' Educational Level**		
No formal education	121	26.6
Primary education(1–8 grade)	147	32.2
Secondary education(9–12 grade)	104	22.8
Diploma and above	84	18.4
**Husband/partner Educational level**		
No formal education	60	14.4
Primary education(1–8 grade)	85	20.4
Secondary education(9–12 grade)	114	27.4
Diploma and above	157	37.8
**Mothers' Occupation**		
Housewife	241	52.8
Public Civil servant	78	17.2
Daily laborer	77	16.8
Merchant	60	13.2
**Average household income**		
≥ 1500 ETB	310	67.9
< 1500 ETB	146	32.1
**Distance to nearby health facility in Minute**		
≤ 30 minutes	409	89.6
> 30 minutes	47	10.4

ETB: Ethiopian Birr

### Exposure to education on Essential Newborn Care

Provision of health information regarding ENBC was reported as poor at the health facility and home visit by HEWs especially on ENBC practices other than breastfeeding and immunization. Of the total study subjects, 254 (68.1%) mothers received education on newborn care during ANC visits at the health facility. Moreover, 289(63.4%) and 224(49.1%) of them received education on breastfeeding and immunization, respectively. Though 429 (94.1%) received a home visit by Health Extension Workers (HEWs), only a few mothers received counseling on thermo-regulation (9.3%) and danger signs to newborn (17.5%) (Table 2).

**Table 2:** Mothers exposed to health education on Essential Newborn Care at the health facility and home visits in Mekelle City, Ethiopia, 2016 (n = 456).

Variables	Frequency	Percentage
**Educated about ENBC during ANC visit**		
Yes	307	67.4
No	149	32.6
Education provided on*		
Breastfeeding	289	63.4
Immunization	224	49.1
Cord care	68	15.0
Danger signs of newborn	58	12.7
Thermo-regulation	23	5.1
Low birth weight	21	4.7
Eye care	16	3.4
**Did you receive home visits by HEW?**		
Yes	429	94.1
No	27	5.9
When was the HEWs visit?*		
During pregnancy	331	72.7
After delivery	368	80.6
Provided information by HEWs*		
Immunization	370	81.3
Breastfeeding	370	81.1
Cord care	184	40.2
Danger signs of newborn	80	17.5
Thermo-regulation	42	9.3
Low birth weight	22	4.9
Eye care	11	2.4

NB:

* Multiple responses were possible; ENBC-Essential Newborn Care; HEW-Health Extension Worker

### Knowledge of postnatal mothers’ on Essential Newborn Care

About 80% of respondents believed that wrapping in a warm dry cloth prevents heat loss from neonate, while 43.9% of the mothers mentioned that mother-baby skin to skin contact prevents hypothermia of neonate after birth. The majority of the respondents (84.5%) believe that the newborn baby should not be nursed in a separate room from his/her mother after birth. Among the participants interviewed, 88.2 percent of them correctly stated that the stump should not be covered or bandaged. Three hundred thirty-five (87.8%) postnatal mothers reported that their newborns were breastfed immediately within one hour after delivery. Ninety-five point seven percent reported that exclusive breastfeeding in the first six months should be provided for all newborn babies. Three hundred seventy five (82.2%) participants responded that reddening of the eyes of the newborn is a sign of the eye infection. The overall knowledge question composite showed that the minimum knowledge score was 7 and the maximum score was 17. Respondents who responded above or equals to 75% of the questions were considered as having good knowledge. In this way, respondents who responded above or equals to 75% of questions were 164 (36.1%) (Table 3).

**Table 3:** Knowledge of postnatal mothers’ on Essential Newborn Care in Mekelle City, 2016 (n = 456).

Knowledge of Essential Newborn Care	N (%)
Keep baby skin to skin contact immediately after delivery	200(43.9)
Keep baby warmth by wrapping dry cloth	366(80.3)
Baby be not nursed in separate room from you after delivery	385(84.5)
The umbilical stump of baby not be covered a cloth/bandage	400(87.8)
Umbilical stump be not soiled	387(84.8)
Breastfeeding a newborn immediately after delivery	404(88.7)
Feed breast milk every 2–3 hours per day	216(47.3)
Exclusive breastfeeding in the first six months	436(95.7)
Feeding colostrum	448(98.3)
A newborn requires vaccination at birth	453(99.4)
Vaccination of newborn prevents disease	451(98.9)
Receive BCG vaccine at birth	277(60.8)
Receive OPV vaccine at birth	398(87.4)
BCG protects a newborn baby from Tuberculosis	186(40.8)
OPV protects a newborn baby from Polio	291(63.8)
Eye discharge is a sign of an eye infection	177(38.8)
Reddening of Eyes is a sign of eye infection	375(82.2)
Swollen eye is a sign of eye infection	272(59.5)
**Good knowledge (answered ≥75% of question correctly)**	**164(36.1)**
**Knowledge about danger signs of newborn**	
Yellowish discoloration of eyes, palms, and soles	48(10.5)
Unable to breastfeed	302(66.2)
Abnormal jerking movement of limbs and eyes	55(12.1)
Difficulty of breathing	179(39.3)
High-grade fever	384(84.3)
A baby cold to touch	44(9.6)
Lethargic baby	62(13.6)
Abdominal distention	25(5.4)
Vomiting /diarrhea	304(66.7)
Crying excessively	84(18.5)
**Good knowledge (know ≥3 danger signs)**	**302(66.2)**

**N.B**: BCG- Bacille Calmette Guerin; OPV- Oral Polio Vaccine

### Practice of postnatal mothers’ on Essential Newborn Care

The first bath was given after 24 hours of birth by 2358 (78.5%) mothers. Three hundred five(66.9%) of participants kept their newborn baby warm by wrapping them with a dry cloth and covering the whole body including head and legs. Exclusive breastfeeding was practiced by 444 (97.4%) mothers. Eye care was practiced by 259 (56.9%) mothers. The application of traditional substances to the eye of the newborn was practiced by 301(67.1%) mothers. The minimum practice score was 3 and the maximum score was 8. Respondents who answered above or equals to the 75% of practice questions were considered as having a good practice. In this way, the overall practice question composite showed that the women who responded above or equals to 75% of practice questions were 370(81.1%) (Table 4).

**Table 4:** Practice of postnatal mothers’ on Essential Newborn Care in Mekelle City, 2016(n = 456).

Essential Newborn Care practice	N (%)
Changed the moist cloths after you dry the baby immediately after delivery	342 (74.9)
Kept newborn warmth by covering all body including the head and legs	305(66.9)
Gave bath for her baby after 24 hours of birth	358(78.5)
Counseled on feeding practices	404(88.5)
Baby fed exclusively breast milk	444(97.4)
Baby fed breast milk every 2–3 hours per day	218(47.8)
The baby received eye ointment immediately after birth	259(56.9)
Not put any substance like Kuhli to the eye of newborn after birth	155(33.9)
**Overall good practice (answered ≥75% of question correctly)**	**370 (81.1)**

### Factors associated with maternal knowledge of Essential Newborn Care

Good knowledge was positively associated with mothers who had some formal education. Living far from the health center, and receiving education about newborn care at ANC visit were also associated with an increased chance of having good ENBC knowledge (Table 5).

**Table 5:** Factors associated with maternal knowledge of Essential Newborn Care in Mekelle City, Tigray, Ethiopia, 2016 (n = 373).

Variables	Knowledge of newborn care (n = 456)^a^		COR(95%CI)	AOR(95%CI)
	Poor: n (%)	Good: n (%)		
**Mothers educational status**				
No formal education	85(70.5)	35(29.5)	1	1
Had a formal education	206(61.6)	129(38.5)	1.54(0.59, 4.02)	2.1(1.01, 4.36)*
**Distance from nearby health institution**				
>30 minutes	20(42.5)	27(57.5)	1	1
≤30 minutes	271(66.4)	137(33.6)	0.38(0.19, 0.78)**	0.32(0.16, 0.65)*
**Information received on ENBC during/after birth**				
No	52(82.5)	11(17.5)	1	1
Yes	239(60.9)	153(39.1)	2.47(0.74, 8.26)	1.62(0.70, 3.73)
**Education received at ANC**				
No	108(72.8)	40(27.2)	1	1
Yes	183(59.6)	124(40.4)	1.92(1.05, 3.48)*	2.14(1.23, 3.71)**

N.B:

^**a**^ weighting factor was used to estimate proportions but not for regression analysis; COR- Crude Odds Ratio; AOR-Adjusted Odds Ratio; 1- reference;

Significant at:

* p-value <0.05;

**p-value<0.01;

*** p-value <0.001

### Factors associated with maternal practices on Essential Newborn Care

Mothers who had some formal education had about 2 times (AOR = 1.94; 95% CI (1.07, 3.50)) higher odds of good practice than those who had no formal education. Married mothers had 3.48 times (AOR = 3.48; 95%CI (1.61, 7.53)) higher odds of having a good ENBC practice than divorced/widowed. Mothers who have been informed of newborn care practices during and after birth had 4.97 times (AOR = 4.97; 95% CI (1.93, 12.76)) higher odds of good practice than those who had no health information about ENBC during ANC visit. Mothers who have good knowledge of ENBC had 2.32 times (AOR = 2.32; 95%CI (1.18, 4.55)) higher odds of good ENBC practice than their poor knowledge counterparts. Mothers who have good knowledge of newborn danger signs (who responded ≥ 3 questions correctly) had 2.43 times (AOR = 2.43; 95%CI (1.21, 4.87)) higher odds of good ENBC practice than their poor knowledge counterparts (Table 6).

**Table 6:** Factors associated with maternal practice on Essential Newborn Care in Mekelle City, Tigray, Ethiopia, 2016(n = 373).

Variables	Newborn care Practice(n = 456) ^a^		COR(95%CI)	AOR(95%CI)
	Poor: n (%)	Good: n (%)		
**Mothers educational status**				
No formal education	34(28.0)	87(72.0)	1	
Had formal education	52(15.5)	282(84.5)	2.13(1.23, 3.68)**	1.94(1.07, 3.50)*
**Marital status**				
Single(widowed/divorced)	15(36.0)	27(64.0)	1	1
Currently married	71(17.1)	343(82.9)	3.15(1.79, 5.53)***	3.48(1.61, 7.53)*
**Information received on ENBC at ANC**				
No	41(27.3)	108(72.7)	1	1
Yes	45(14.7)	262(85.3)	5.69(2.15, 15.07)***	**1.94(0.84, 4.50)**
**Information received on ENBC practices during /after birth**				
No	31(48.5)	32(51.5)	1	1
Yes	55(14.1)	338(85.9)	5.69(2.89, 11.20)***	4.97(1.94, 12.76)**
**Knowledge of newborn care**				
Poor(<75% of questions)	73(25.1)	218(74.9)	1	1
Good(≥75% of questions)	13(7.7)	152(92.3)	3.58(2.00, 6.43)***	2.32(1.18, 4.56)*
**Knowledge of danger signs**				
Poor(<3 questions responded correctly)	50(32.7)	104(67.3)	1	1
Good (≥3 questions)	36(11.8)	266(88.2)	3.33(1.53, 7.25)**	2.43(1.21, 4.87)*

NB:

^**a**^ weighting factor was used to estimate proportions but not for regression analysis; ANC-Antenatal Care; ENBC-Essential Newborn Care; 1- reference,

Significant at p-value:

* <0.05;

**<0.01;

***<0.001

## Discussion

This study showed that 36.1% of the respondents have good knowledge of ENBC. The current finding is lower than the finding of a community-based study conducted in Ethiopia (80.4%) [20]. This difference might be due to the degree of acceptance of the health extension program by the community and the individual competencies of professionals in both settings. Poor quality postnatal service, counseling and inadequate home visits can contribute to the current problem. If the mother has poor knowledge, they are more likely to use harmful traditional practices which may affect the health and development of newborns. This may have an impact on the current global and national plan to reduce neonatal mortality by 2030 [21]. In addition, community acceptance, individual competencies of urban health extension professionals and lack of supportive systems are evidenced as challenges in the implementation of the urban health extension program [22, 23]. Therefore, strengthening the program in a way that conforms to the community, improving the capacity of urban health extension workers and creating supportive systems are needed to improve the awareness of urban communities regarding newborn care.

In the current study, the overall good ENBC practice was 81.1%, which is comparable with other studies conducted in Ethiopia in Gulomekada District (92.9%) [20]. In contrast, the current study result was higher than a similar study conducted in Ethiopia (in rural Awabel District), which revealed that 23.1% of the postnatal mothers have a good ENBC practice [19]. This might be due to the differences in study setting in which participants of the current study were from an urban population that may have better access to health information and service.

The majority(89.8%) of participants breastfed their newborn immediately (within one hour) after birth. This is slightly higher than previous study done in Mekelle City that reported 77.9% of mothers initiated breast milk timely for their newborn[24]. This could be due to improvements in the awareness of mothers due to increase in access to the maternal and child health services through time. Regarding practice on thermal-regulation, 78.3% of the mothers bathed their baby after 24 hours of age, which is much higher than previous studies conducted in Ethiopia Awabel District (34.4%) [19], and in four regions of Ethiopia (25.3%) [18].

The educational status of the mothers was found to be positively associated with knowledge and practice on ENBC. In line with this finding, it has been identified that higher levels of parental education have a significant impact on the level of knowledge about newborn care, danger signs and their health seeking behavior [20, 25, 26]. Easy access to the health facility is expected to improve community awareness towards healthy behaviors. However, the current finding showed that respondents who lived far from the health facility (more than 30 minutes to reach on foot) had better knowledge compared to mothers who lived near a health facility. This might be because women that live near health facility may not be keen to know about newborn care as they can easily access the health facility at the time when they need help.

Married women had 3.48 times higher odds of having a good practice than others (divorced/widowed) that is consistent with the finding of a similar study in Ethiopia [20]. Getting information on ENBC during ANC visit was found to have a positive association with ENBC practice. Likewise, health information about ENBC during delivery and postpartum had a positive association with ENBC practice. The result of the current study was also similar to a study conducted in Ethiopia [19], which showed postpartum women who were informed on newborn care practices during delivery and postpartum were more likely to practice good ENBC. Mothers who had good knowledge of neonatal danger signs were more likely to have good knowledge of ENBC than those who did not have. Moreover, women who had good knowledge of ENBC had 2.32 times higher odds of good practice than those women who didn’t, which is consistent with the finding of a community-based study conducted in Ethiopia [20].

Though Ethiopia has made tremendous efforts to increase the access and quality of health services for its people [8], the current findings showed that the provision of health information on ENBC was reported as low at both ANC visit and at home visits by HEWs especially on ENBC practices other than breastfeeding and immunization. Consequently, significant proportions of mothers had poor knowledge and practice of ENBC. We, therefore, recommend that the maternal and child health services should be further strengthened at both health facility and community levels through improving the capacity of health professionals and urban HEWs regarding ENBC.

As this study is conducted at the community level, it has the opportunity to collect the opinion of participants at grass root level and helps to devise mechanisms to improve the services to the satisfaction of the community. This is possible through the identification of knowledge gaps and poor practice of ENBC. The reliable estimates were made because statistical adjustments for the complex survey design were done at both design and analysis stages. However, the study was based on reported rather than observed knowledge and practice towards newborn care which could be the possible limitation of this study. In addition, due to the cross-sectional nature of the survey, it could be difficult to see the causal relationship between the independent and the dependent variables. The observed associations between the independent and dependent variables could be confounded by variables that were not measured in this study. The study was based on the sample with the assumption of homogeneity among the clusters; this may not be true in real situations.

## Conclusions

A substantial number of postpartum mothers had poor knowledge and practice of ENBC and newborn danger signs in Mekelle city. Marital status, personal level of education, provision of health education on ENBC at ANC and during or after delivery of newborn care were found to be positively associated with knowledge and practice of postpartum mothers with ENBC. Therefore, improving quality of and access to, perinatal care services and home visits using the urban health extension workers at the community level should be encouraged.

## Supporting information

S1 File The study data. (SAV)

## Abbreviations

ANC: Antenatal Care
BCG: Bacille Calmette Guerin
ENBC: Essential Newborn Care
HEW: Health Extension Worker
OPV: Oral Polio Vaccine
WHO: World Health Organization
